# Special Anatomy and Histology

**Published:** 1843-10-14

**Authors:** 


### BIBLIOGRAPHICAL NOTICES.

*Special Anatomy and Histology.* By William E. Horner, M. D. Professor of Anatomy in the University of Pennsylvania, &c. &c. *In two Volumes. Sixth Edition.* Philadelphia: Lea and Blanchard. 1843. 8vo. pp. 536—547.

Since the first edition (1826) of this work, Anatomy has made immense progress, and General Anatomy or Histology, has been almost remodeled by means of the microscope. To meet the actual condition of the science, Professor Horner has recast a portion of his treatise, and made considerable additions in the other parts. Two-thirds of the Introductory Chapter on Histogeny is new; that on Glandular Structure has been re-written; and the one devoted to the blood has been essentially modified. In all, two hundred pages of manuscript have been introduced. The character of the work is too well known to need any comments from us. Our remarks will refer only to the present edition. It may be objected to it, perhaps, that it is still too conservative, and that there is too little disposition to admit new descriptions and new views, especially in the Histological sections. When we consider that the science of General Anatomy is at present in a transition state, and that what is positively affirmed to-day, is as positively denied to-morrow, it becomes both the teacher and writer on Anatomy to pause, before he gives currency to doctrines of doubtful correctness, more especially in a text-book—very frequently the only authority of both the student and practitioner. If we were disposed to be critical we should say that the Chapter on "Chemical Composition" more properly belongs to a Physiological, than an Anatomical treatise; but this is a matter of opinion and taste.

In treating of the various organic proximate elements, Professor Horner has adopted, in common with all recent authorities, the analyses of Mulder. Several of the neutral azotized materials of organization have very recently been examined by MM. Dumas and Cahours, the analysis being conducted on a very large scale, and a much closer agreement being obtained than by any previous observer—a satisfactory evidence of the accuracy of the method employed. The average composition of five different kinds of animal albumen was a follows:

| | |
|---|---|
| Carbon, . . . . . | 53.42 |
| Hydrogen, . . . . . | 7.19 |
| Azote, . . . . . . | 15.74 |
| Orygen, Sulphur, and Phosphorus, | 23.65 |
| | 100.00 |

In no instance was there a departure from this average indicated, to the amount of more than .1, or at most .2 per cent.

From the analyses of MM. Dumas and Cahours, it would appear to be positively determined that fibrin so far as regards the four elements—Carbon, Azote, Hydrogen, and Oxygen—from being identical with albumen, very essentially differs from it. The following are the results of these analyses, which certainly present a very remarkable accordance.

| | Blood of | | | | | | | |
|---|---|---|---|---|---|---|---|---|
| | Sheep | Calf | Ox | Horse | Dog | Dog A | Dog B | Man |
| Carbon, . . | 52.8 | 52.5 | 52.7 | 52.67 | 52.74 | 52.77 | 52.57 | 52.78 |
| Hydrogen, . | 7 0 | 7 0 | 7.0 | 7.0 | 6.92 | 6.95 | 7.07 | 6.96 |
| Azote, . . . | 16.5 | 16.6 | 16.6 | 16.63 | 17.72 | 16.51 | 16.55 | 16.78 |
| Oxygen, &c. | 23.7 | 24.0 | 23.7 | 23.70 | 23.62 | 23.77 | 23.81 | 23.48 |

The proportion of Carbon, we find, is .7 per cent. less in fibrin than in albumen, whilst the proportion of Azote is from .8 to .9 per cent. more. A correct idea of the elementary composition of fibrin may be formed, if we consider it a composition of casein, or albumen, and ammonia. The same observers have found a vegetable fibrin equally distinct from vegetable albumen. This view of the chemical composition of albumen and fibrin would derive additional weight if we consider the difference in their physiological properties which, are still more marked. Albumen may be regarded as the raw material from which the yarn or fibrin is elaborated to be subsequently converted into the fabric.

At page 56 of the Chapter on Histogeny, we find this sentence:

"From the state then of nucleated Cell [s], as described in the foregoing pages, all the tissues may be traced as they exist in the perfect and mature animal." From this doctrine we must record our dissent.

If the clot formed by the coagulation of fibrin out of the body, be examined carefully under the microscope, it will be found to consist of a complete interlacement of fibres; and the firmer the coagulation, the more slowly it has occurred. When fibrin is poured out on a living surface, its tendency to assume this regular arrangement is still more marked. Now there is no doubt but that several of the elementary tissues of the animal body are formed in this way. This structure is most satisfactorily seen, and in its simplest form, in the membrane which forms the basis of the egg-shell, and encloses the white. If you soak for some time the membrane in water, you can separate it into several layers, each one presenting a beautiful *matted* appearance, and composed of an interlacement of fibres. The structure of the egg-shell is identical, if its calcareous matter be removed by a weak acid. This may be regarded as the type of the fibrous tissues, with this exception: that the one being temporary, there is no provision for renovation or growth—no trace of nutrient vessels, whilst the other is permanent, and its preservation provided for by a supply of blood-vessels.

